# The Effect of Piceatannol from Passion Fruit (*Passiflora edulis*) Seeds on Metabolic Health in Humans

**DOI:** 10.3390/nu9101142

**Published:** 2017-10-18

**Authors:** Munehiro Kitada, Yoshio Ogura, Hiroko Maruki-Uchida, Masahiko Sai, Taeko Suzuki, Keizo Kanasaki, Yuna Hara, Hiromi Seto, Yuka Kuroshima, Itaru Monno, Daisuke Koya

**Affiliations:** 1 Department of Diabetology and Endocrinology, Kanazawa Medical University, 1-1 Daigaku, Uchinada, Ishikawa 920-0293, Japan; namu1192@kanazawa-med.ac.jp (Y.O.); tsuzuki@kanazawa-med.ac.jp (T.S.); kkanasaki@kanazawa-med.ac.jp (K.K); naipicrc@kanazawa-med.ac.jp (Y.K.); imonno@kanazawa-med.ac.jp (I.M.); koya0516@kanazawa-med.ac.jp (D.K.); 2 Division of Anticipatory Molecular Food Science and Technology, Medical Research Institute, Kanazawa Medical University, 1-1 Daigaku, Uchinada, Ishikawa 920-0293, Japan; 3 Research and Development Department, Health and Wellness Headquarters, Morinaga and Company Limited, 2-1-1 Shimosueyoshi, Tsurumi-ku, Yokohama 230-8504, Japan; h-uchida-ji@morinaga.co.jp (H.M.-U.); m.sai@miobs.com (M.S.); 4 Division of Clinical Laboratory, Kanazawa Medical University Hospital, 1-1 Daigaku, Uchinada, Ishikawa 920-0293, Japan; yuna-h@kanazawa-med.ac.jp (Y.H.); hi-seto@kanazawa-med.ac.jp (H.S.)

**Keywords:** piceatannol, an analogue of resveratrol, metabolic health, insulin sensitivity

## Abstract

Animal studies have shown the beneficial effects of piceatannol on metabolic health; however, there is a lack of human studies designed to examine these effects. The objective of this study was to investigate the effects of piceatannol on metabolic health in humans. This randomized, placebo-controlled study was conducted on 39 subjects, including 10 overweight men and 9 overweight women (BMI ≥ 25), as well as 10 non-overweight men and 10 non-overweight women (BMI < 25). Subjects received piceatannol (20 mg/day) or placebo capsules for eight weeks in a random order. The primary outcome was the effect of piceatannol on glucose-metabolism, including insulin sensitivity. The secondary outcomes were the effects on other parameters, including blood pressure (BP), heart rate (HR), endothelial function, lipids, inflammation, oxidative stress, mood status, and Sirt1 and phospho-AMP-activated kinase (p-AMPK) expression in isolated peripheral blood mononuclear cells (PBMNCs). Supplementation with piceatannol in overweight men reduced serum insulin levels, HOMA-IR, BP and HR. Other groups, including non-overweight men, as well as overweight and non-overweight women, showed no beneficial effects on insulin sensitivity, BP and HR. Furthermore, piceatannol is not associated with other data, including body weight (BW), body composition, endothelial function, lipids, inflammation, oxidative stress, mood status, and Sirt1/p-AMPK expression in PBMNCs. In conclusion, supplementation with piceatannol can improve metabolic health, including insulin sensitivity, BP and HR, in overweight men.

## 1. Introduction

Obesity and metabolic syndrome (MS) complicate metabolic derangement, including insulin resistance, hypertension, and dyslipidemia [1], and are also closely related to aging [2,3]. Therefore, maintaining metabolic health may have beneficial effects for the prevention of metabolic derangement and for anti-aging. Basically, improvement of lifestyle, especially diet therapy, such as calorie restriction (CR), is important for the prevention of obesity, MS and cardiovascular disease, which is related to MS based on insulin resistance [4,5]. However, it is not easy to continue CR for a long time, and therapeutic options are still insufficient; therefore, novel treatment modalities, including functional foods, have been investigated. Polyphenolic compounds, such as resveratrol, have attracted attention over the past few decades because previous reports have shown that resveratrol treatment in mice fed a high calorie diet consistently improved various health parameters, including glucose metabolism, endurance and survival [6,7,8,9,10], and these compounds may act as a CR mimetic. In a study on humans, Timmers et al. reported that resveratrol supplementation for 30 days in obese humans showed CR-like effects, such as increased insulin sensitivity, reduced blood pressure (BP), improvement of muscle mitochondrial respiration, and activation of Sirt1 and AMP-activated kinase (AMPK) [11]. Piceatannol is a polyphenolic stilbene phytochemical and is known to be a hydroxylated analog of resveratrol. Piceatannol shows activity similar to resveratrol [12]; however, because its levels in plants such as grapes, and wines, are significantly lower than those of resveratrol [13], it has received far less research attention. Matsui et al. previously found that passion fruit (Passiflora edulis) seeds have a high piceatannol content [14], and several reports have shown that piceatannol has various properties, such as Sirt1 induction activity [15], a vasorelaxant effect [16], upregulation of endothelial nitric oxide synthase (eNOS) expression [17], promotion of collagen synthesis [14], inhibition of melanogenesis [14], and protection of the skin from ultraviolet B (UVB) irradiation [18]. Piceatannol shows much higher activity than resveratrol in some cases [14,17]; however, whether these effects of piceatannol apply to humans remains uncertain. In this study, we aimed to investigate the effects of piceatannol in humans on metabolic health, including glucose metabolism, BP, heart rate (HR), endothelial function, lipids, inflammation, and oxidative stress. Furthermore, we assessed the differences of the effects of piceatannol among overweight and non-overweight men and women.

## 2. Materials and Methods

### 2.1. Subjects and Study Design

Participants were recruited through advertisements on local posters. Men or women who were 20–70 years old were eligible, and inclusion was ultimately based on a normal physical examination, including routine clinical biochemistry data. The exclusion criteria included overt endocrine disease, renal disease, hepatic disease, heart disease, malignant disease, anemia, alcohol abuse, smoking, taking prescription medicine and planned lifestyle changes. We enrolled 39 participants, including 10 non-overweight (Body mass index (BMI) < 25 kg/m^2^) men and 10 non- overweight women, as well as 10 overweight (BMI ≥ 25 kg/m^2^) men and 9 overweight women, in this study.

The study was an investigator-initiated randomized, double-blinded, placebo-controlled trial. Subjects were randomly assigned (1:1) to treatment for 8 weeks with capsules containing 5 mg of piceatannol, which was purified from passion fruit seed extract using gamma-cyclodextrin (Purity 81.4%) or containing dexitrin, a placebo (2 capsules of piceatannol with 5 mg/capsule, twice daily for a total of 20 mg/day or 2 capsules of placebo (dextrin) 5 mg/capsule, twice daily). The capsules containing piceatannol and placebo with cellulose and other diluents were provided by Morinaga and Company Limited, and packaging and labeling were also performed by Morinaga and Company Limited. Randomization and blinding was performed by the pharmacy at Kanazawa Medical University Hospital. The randomization code was revealed once all the predefined data was recorded. During the trial period, subjects were instructed to abstain from using nutritional supplements and consuming food suspected to contain polyphenols in significant amounts. Furthermore, the importance of maintaining their normal way of life was underscored. Compliance, defined as the proportion of tablets ingested relative to the intended number, was calculated when participants returned the remaining tablets during the final examination.

### 2.2. Overall Visits and Interventions

Examinations were performed on 3 consecutive days at baseline, as well as after 4 and 8 weeks of treatment, with the same equipment and by the same physicians on both occasions. When completing the physical examination, including routine clinical biochemistry data at baseline, capsules were provided and subjects were instructed to initiate capsule consumption in the evening and twice daily until the overnight fast before the examination day at week 4. At week 4, potential adverse events were recorded and fasting blood samples were taken for safety purposes. Additionally, participants visited the hospital on the examination day at week 8 in the morning after overnight fasting, and then blood samples were collected 15 min after taking the capsules for pharmacological purposes.

### 2.3. Ethical Approval

Patients were given detailed explanations of the study protocol. Informed consent was obtained from each patient. The study protocol was approved by the Regional Committee on Health Research Ethics and the Ethical Committee of Kanazawa Medical University. The trial was registered with the University Hospital Medical Information Network (UMIN No. 000018397).

### 2.4. General Measurements

Body weight (BW) and body composition were measured using In Body (Biospace Japan, Inc., Tokyo, Japan) with the participants lightly clothed. In addition, BP and HR were measured in a sitting position after resting for 5 min. Routine biochemistry and physical examinations were also performed at screening to investigate the presence of exclusion criteria.

### 2.5. Blood Sample Analysis

Routine biochemistry (creatinine (Cr), uric acid (UA), aspartate aminotransferase (AST), alanine transaminase (ALT), and γ-glutamyl transpeptidase (γ-GTP)) were analyzed continuously throughout the study. HbA1c and glycated albumin were measured using an automated analyzer, HLC-723^®^ G11 (Tosho Co., Ltd., Tokyo, Japan). Serum low-density lipoprotein-cholesterol (LDL-C) and high-density lipoprotein-cholesterol (HDL-C) levels were measured using enzymatic methods (QUALIGENT^®^ HDL-C and QUALIGENT^®^ LDL-C, Sekisui Medical Co., Ltd., Tokyo, Japan). Serum triglyceride (TG) levels were measured using enzymatic assays (Kyowa Medex, Co., Ltd., Tokyo, Japan). Free fatty acids (FFAs) were measured by a commercially available kit (Wako Chemicals, Neuss, Germany). The estimated glomerular filtration rate (eGFR) was calculated as 194 × serum creatinine^−1.094^ × age^−0.287^ in males and as 194 × serum creatinine^−1.094^ × age^−0.287^ × 0.739 in females [19]. Plasma glucose was measured in duplicate immediately after sampling on an YSI 2300 Stat Plus (YSI, Inc., Yellow Springs, OH, USA). AST, ALT, γ-GTP and UA were analyzed at the University Hospital Department of Clinical Biochemistry using standard methods. Insulin was analyzed using a time-resolved immunofluorometric assay (AutoDELFIA Insulin kit, catalog no. B080–101, PerkinElmer, Turku, Finland). Homeostasis model assessment–insulin resistance (HOMA-IR) was calculated using the standard formula based on fasting glucose and insulin. Serum for analyses of diacron reactive oxygen metabolite (dROM), biological antioxidant potential (BAP), asymmetric dimethylarginine (ADMA), interleukin-6 (IL-6), and high sensitivity C-reactive protein (hsCRP) was frozen and stored at −80 °C, after being drawn and centrifuged, until the time of analysis. Oxidative stress was evaluated by the d-ROMs test, which is a simple assay marketed to analyze the total amount of hydroperoxides in serum via the Fenton reaction. The BAP test, which is based on the ability of a plasma blood sample to reduce ferric ions to ferrous ions as a FRAP assay (ferric reducing ability of plasma), was performed to assess antioxidant capacity. The BAP test and dROM test were conducted using a specially designed photometer in conjunction with the FRAS4 system (Health & Diagnostic Ltd., Co., Parma, Italy) [20]. Serum IL-6 was measured by Human IL-6 CLEIA (Chemiluminescent Enzyme Immuno Assay) Fujirebio (Tokyo, Japan), and hs-CRP was measured by a nephelometry method using N-Latex CRPII (Siemens Healthineers, Tokyo, Japan). Serum ADMA was evaluated by HPLC (high-performance liquid chromatography) (JASCO Corporation, Tokyo, Japan).

### 2.6. Flow Mediated Dilation (FMD)

FMD was measured at baseline and after 8 weeks by an experienced technician using the UNEX EF38G (UNEX Corporation, Nagoya, Japan). The technician was blinded to the study groups. The detailed protocol and methodology have been previously described in detail [21,22]. All measurements were performed under fasting conditions in the morning in a temperature-controlled room (25 °C). After resting for 15 min, a pressure cuff was placed on the forearm to capture baseline images of the brachial artery using high-resolution ultrasound. Then, the cuff was inflated and kept at 50 mmHg above the systolic BP to occlude the brachial artery. After 5 min, the cuff was released and an image of the brachial artery was captured. The diameters of the brachial artery on the pre- and post-hyperemia images were used to calculate changes in FMD according to the following formula: (FMD (%) = (maximum diameter − diameter at rest) × 100/diameter at rest).

### 2.7. Sirt1 Expression and p-AMPK Expression in Isolated Peripheral Blood Mononuclear Cells (PBMNCs)

PBMNCs were collected from 20 mL of heparinized blood and isolated using Histopaque-1077 (Sigma-Aldrich, St. Louis, MO, USA) as previously reported [23]. PBMNCs were washed three times with Phosphate Buffered Saline (PBS) (without magnesium and calcium) and suspended in Radioimmunoprecipitation (RIPA) buffer with a protease inhibitor cocktail (Sigma-Aldrich, St. Louis, MO, USA) for protein extraction.

The samples from isolated PBMNCs were homogenized in ice-cold RIPA buffer. Samples of protein solutions were used for western blotting. These samples were separated on 15% SDS-PAGE (Sodium dodecyl sulfate-polyacrylamide gel electrophoresis) gels and transferred to a polyvinylidene difluoride filter (Immobilon; Millipore, Bedford, UK). After blocking the samples with 5% milk, the filter was incubated overnight with an anti-phospho(p)-AMPK α (Thr 172), AMPKα, β-actin (Cell Signaling Technology Inc., Danvers, MA, USA), and Sirt1 (Millipore, Bedford, MA, USA) antibodies at 4 °C [23]. The filter was then incubated with the appropriate Horseradish peroxidase (HRP)-conjugated secondary antibodies (Amersham, Buckinghamshire, UK), and the bands were detected by enhanced chemiluminescence (Amersham, Buckinghamshire, UK).

### 2.8. Evaluation of Mood States, Weariness and Stiffness

The Profile of Mood States (POMS) is a well-validated questionnaire of mood states and their fluctuations in clinical research [24,25]. We administered the Japanese version of the POMS test (Kaneko Shobo Co., Tokyo, Japan) [25]. Participants rated sixty-five adjectives in terms of how much they had felt each mood state in the past week using a five-point scale from “not at all” to “extremely”. Scores from these sixty-five items were combined to give six global scores of “tension”, “depression”, “anger”, “fatigue”, “confusion” and “vigor”. A total mood disturbance score was also calculated by adding the scores from the first five of these global scores and subtracting ‘’vigor”.

The visual analogue scale (VAS) is a simple assessment tool consisting of a 10 cm line with 0 on one end representing no symptoms and 10 on the other representing the highest intensity ever experienced, which a patient marks to indicate the severity of a specific manifestation. This scale was used to evaluate weariness and stiffness in this study [26].

### 2.9. Statistical Analysis

Data are presented as the means ± standard deviation (SD) unless otherwise indicated. The results obtained at baseline and after 8 weeks of piceatannol supplementation or placebo, as well as changes within a group, were compared using a paired *t*-test, Wilcoxon Signed-rank Test or Mann-Whitney *U*-test. The results were statistically significant if *p* < 0.05 (two sided). Statistical analysis was performed using SAS 9.4 (SAS Institute, Cary, NC, USA). The statistic analyze was performed by Kureha Special Laboratory Co., Ltd. (Tokyo, Japan).

## 3. Results

### 3.1. Baseline Characteristics

A total of 39 subjects, which included 20 men and 19 women, participated in this study. Baseline characteristics in all subjects in eight groups, including non-overweight men treated with placebo (*n* = 5) or piceatannol (*n* = 5), overweight men treated with placebo (*n* = 5) or piceatannol (*n* = 5), non-overweight women treated with placebo (*n* = 5) or piceatannol (*n* = 5) and overweight women treated with placebo (*n* = 4) or piceatannol (*n* = 5), are shown in Table 1. BMI in subjects who were treated with piceatannol was more than 25 (≥25) and less than 30 (<30). At baseline, the fasting serum insulin and TG levels were significantly higher in overweight men treated with piceatannol than in overweight men treated with placebo (Insulin; piceatannol 8.3 ± 1.9, placebo 6.1 ± 1.0 μU/mL, *p* = 0.0367, TG; piceatannol 181.0 ± 66.4, placebo 98.6 ± 42.9 mg/dL, *p* = 0.0367). The HbA1c levels at baseline were significantly lower in non-overweight men treated with piceatannol than in non-overweight men treated with placebo (Piceatannol 5.2 ± 0.2, placebo 5.6 ± 0.2%, *p* = 0.0439). There was no statistically significant difference for the other baseline characteristics, including age, BW, BMI, BP, HR, glucose/lipid/liver and kidney function-related data, history of smoking and alcohol intake, among each of the two intervention groups, placebo and piceatannol.

### 3.2. Changing BW, BMI, Body Composition, BP and HR

**Figure 1:**
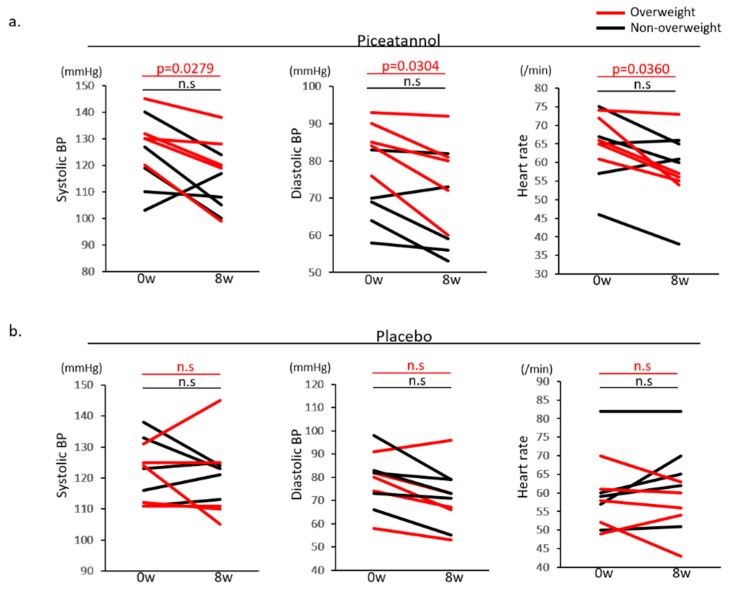
Systolic blood pressure (BP), diastolic BP and heart rate (HR) in overweight (shown as a red line) or non-overweight (shown as a black line) men at baseline and 8 weeks after piceatannol supplementation (**a**) or placebo (**b**). *p* values between two groups are shown, and n.s. denotes no significance.

In Table 2, BW, BMI, and body composition, including % fat, muscle weight and visceral fat area, which were evaluated by In Body, were not significantly different between the groups treated with placebo and piceatannol. There was no significant difference in systolic and diastolic BP and HR between groups treated with placebo and piceatannol in non-overweight men and women or in overweight women (Table 2a,b). In overweight men, piceatannol supplementation for 8 weeks significantly reduced systolic and diastolic BP, HR (systolic BP 131.4 ± 8.9 (at baseline)→120.8 ± 14.4 mmHg (after 8 weeks), *p* = 0.0279, diastolic BP 85.6 ± 6.5 (at baseline)→77.0 ± 11.9 mmHg (after 8 weeks), *p* = 0.0304, HR 67.6 ± 5.3 (at baseline)→59.0 ± 7.9/min (after 8 weeks), *p* = 0.036) (Table 2a and Figure 1a). However, there was no significant change in overweight men treated with placebo after 8 weeks (Table 2b and Figure 1b).

### 3.3. Change in Glucose Metabolism-Related Data

**Figure 2:**
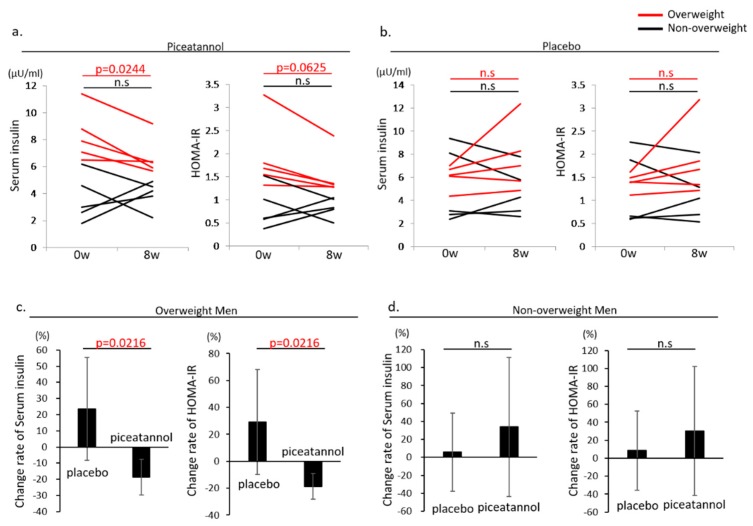
Serum insulin levels and HOMA-IR in overweight (shown as a red line) or non-overweight (shown as a black line) men at baseline and 8 weeks after piceatannol supplementation (**a**) or placebo (**b**). *p* values between two groups are shown, and n.s. denotes no significance. Change rate of serum insulin levels and HOMA-IR in overweight (**c**) or non- overweight (**d**) men at baseline and after 8 weeks of piceatannol supplementation or placebo. *p* values between two groups are shown, and n.s. denotes no significance.

For the data on glucose metabolism shown in Table 3a–c, piceatannol supplementation for 8 weeks significantly decreased fasting serum insulin levels in overweight men (8.3 ± 1.9 (at baseline)→6.7 ± 1.4 µU/mL (after 8 weeks), *p* = 0.0244) (Table 3a and Figure 2a). However, in overweight men treated with placebo, serum insulin levels showed no change after 8 weeks (6.1 ± 1.0 (at baseline)→7.7 ± 2.9 µU/mL (after 8 weeks), *p* = 0.1918) (Table 3a and Figure 2b). In addition, the change rate for both serum insulin and HOMA-IR significantly decreased in overweight men treated with piceatannol, which indicated that there was an improvement in insulin sensitivity (Serum insulin; placebo 23.7 ± 31.8%, piceatannol −18.8 ± 11.2%, *p* = 0.0216 and HOMA-IR; placebo 31.4 ± 39.7%, piceatannol −17.2 ± 11.5%, *p* = 0.016) (Figure 2c). In non-overweight men, the change rate of both serum insulin and HOMA-IR showed no change between placebo and piceatannol supplementation (Figure 2d). Piceatannol supplementation for 8 weeks in other groups did not affect the levels of fasting serum insulin and HOMA-IR. In addition, in the non-overweight women group, piceatannol supplementation significantly reduced the levels of glycated albumin from baseline (14.4 ± 1.6 (at baseline)→13.9 ± 1.4% (after 8 weeks), *p* = 0.0118) Table 3b), despite no changes in serum insulin and HOMA-IR.

### 3.4. Changes in Liver and Renal Function Tests and Lipid Data

The results of liver function tests, such as AST, ALT and γ-GTP, and serum Cr, eGFR, and serum UA, as well as lipid data, including the total cholesterol, HDL-C, LDL-C, TG and FFA levels, are shown in Table 4a,b. All data showed no significant differences in any of the groups treated with placebo or piceatannol.

### 3.5. Changes in Endothelial Function, Inflammation and Oxidative Stress

Endothelial function, which was evaluated by FMD; serum levels of ADMA, which is an inhibitory molecule of eNOS; inflammatory markers, including serum hsCRP and IL-6; oxidative stress and anti-oxidative capacity, such as dROM and BAP, also showed no significant differences in any of the groups treated with placebo or piceatannol, and the results are shown in Table 5a,b.

### 3.6. Changes in Sirt1 and p-AMPK Expression in Isolated PBMNCs

Supplementation with piceatannol for 8 weeks did not change Sirt1 expression, which was evaluated by western blotting in isolated PBMNCs. Additionally, p-AMPK expression in isolated PBMNCs was not significantly changed by piceatannol supplementation (Supplemental Figure S1).

### 3.7. Changes in Mood Status

We evaluated mood status using the POMS and VAS tests. Supplementation with piceatannol for 8 weeks did not change the mood status-related parameters from the baseline (Supplemental Table S1).

### 3.8. Safety and Adverse Events

The rates of adverse events were low in both groups treated with piceatannol or placebo, and there were no statistically significant differences in adverse events reported from participants in either treatment group compared to the placebo group. Adverse events are listed in Supplemental Table S2, and there were no severe adverse events in this study.

## 4. Discussion

In this study, our data indicated that piceatannol supplementation in overweight men improved insulin sensitivity, which was evaluated by serum insulin levels and HOMA-IR. Additionally, piceatannol supplementation significantly reduced BP and HR in overweight men. However, other groups, including non-overweight men, as well as overweight and non-overweight women, did not show the beneficial effects on insulin sensitivity, BP and HR that were observed after supplementation with piceatannol in overweight men. Furthermore, piceatannol was not associated with other data, including BW, body composition, endothelial function, lipid profiles, inflammation, oxidative stress, mood status, and Sirt1 and p-AMPK expression in isolated PBMNCs.

Piceatannol is a phytochemical that is present in large amounts in passion fruit (Passiflora edulis) seeds [14] and is an analog of resveratrol. Previous reports showed that piceatannol has various effects, such as improvement of hyperglycemia. Uchida-Maruki et al. demonstrated that piceatannol or a passion fruit seed extract reduced blood glucose levels in mice consuming a high-fat diet or db/db mice without changing BW and visceral fat weight [27]. Minakawa et al. also reported that piceatannol promoted glucose uptake, AMPK phosphorylation and glucose transporter4 (GLUT4) translocation in cultured L6 myotubes in the absence of insulin. Furthermore, piceatannol suppressed rises in blood glucose levels at early stages and improved impaired glucose tolerance at later stages in db/db mice [28]. In addition, Zhang et al. demonstrated that dietary polyphenols, such as resveratrol and piceatannol in mice with a high-fat diet, lowered postprandial hyperglycemia, indicating that inhibition of intestinal α-glucosidase activity may be a potential mechanism contributing to their anti-diabetic properties [29]. Therefore, piceatannol may achieve an anti-diabetic effect through multiple mechanisms in animal models. However, the effects of piceatannol in humans have never been reported. In this study, we showed that supplementation with piceatannol for 8 weeks led to an improvement in insulin sensitivity, which was evaluated by fasting serum insulin and HOMA-IR, in overweight men. Additionally, in non-overweight women, piceatannol reduced serum glycated albumin levels after 8 weeks. This data indicates that piceatannol may have anti-diabetic properties in humans. It is still unclear why piceatannol only improved insulin sensitivity in overweight men, and gender difference on the effect of piceatannol is also unclear, as well. However, fat weight and visceral fat area at the baseline in overweight women treated with piceatannol at baseline were significantly higher than those of overweight men treated with piceatannol (*p* < 0.01 vs. overweight women). In addition, skeletal muscle weight at the baseline in overweight men treated with piceatannol was significantly heavier than that of overweight women treated with piceatannol (*p* < 0.01 vs. overweight men). However, it is unclear whether the differences in body composition, including fat and skeletal muscle weights, between overweight men and women treated with piceatannol may be related to the effect of piceatannol on insulin sensitivity. Furthermore, there is a limitation to understanding the effect of piceatannol on detailed glucose metabolism because we only evaluated fasting serum insulin and blood glucose levels, and calculated HOMA-IR, HbA1c and serum glycated albumin. We could not perform a glucose tolerance test or a glucose cramp test.

In addition to the improvement in insulin sensitivity, BP and HR were significantly reduced by piceatannol for 8 weeks in overweight men, which suggested that there is a beneficial effect of piceatannol on the vasculature [30]. Previous reports have shown that piceatannol protects vascular function and exerts anti-oxidative stress and anti-inflammation [16,17,18,31]. Endothelial dysfunction is characterized by impaired endothelium-dependent vasorelaxation and represents an early step in the pathogenesis of atherosclerosis [32]. The known mechanisms of atherosclerosis related to endothelial dysfunction include impaired NO production from eNOS and defects in its signaling pathway, increased oxidative stress, and inflammation. Piceatannol may protect vascular function through the improvement in eNOS, as well as the reduction of oxidative stress and inflammation. Kinoshita et al. demonstrated that piceatannol increased eNOS expression in cultured endothelial cells [16]. In addition, piceatannol improved endothelial function through the activation of dimethylarginine dimethylaminohydrolase (DDAH), which is an enzyme that degrades the natural inhibitor of eNOS, ADMA [33]. Nguyen et al. also reported that piceatannol inhibited arginase activity and reciprocally increased NO generation by increasing eNOS Ser1177 phosphorylation and eNOS dimer stability, which resulted in an improvement in vascular function [34]. Furthermore, Jeong et al. reported that piceatannol had a protective effect against palmitic acid (PA)-induced endothelial dysfunction by inducing NF-E2-related factor-2 (Nrf2)-dependent expression of hemeoxigenase-1 (HO-1), which inhibits PA-induced inflammation, oxidative stress and reduction of eNOS activity [35]. Although piceatannol protects vascular function through the induction and activation of eNOS, anti-oxidative stress and anti-inflammation, as described above, there are no reports regarding the effects of piceatannol on BP or HR. In this study, our data showed that piceatannol reduced BP and HR in overweight men; however, piceatannol did not affect endothelial function, inflammation and oxidative stress, which were evaluated by FMD, inflammatory and oxidative stress markers in this study. It is generally accepted that the rostral ventrolateral medulla (RVLM) plays a pivotal role in the regulation of vascular tone and the maintenance of BP. It is a key region for the control of sympathetic drive to the periphery. The activity of RVLM neurons can dominate peripheral vascular tone and arterial BP [36]. Ma et al. previously demonstrated that a microinjection of resveratrol into the RVLM inhibits BP, HR, and renal sympathetic nerve activity (RSNA) [37]. It is unknown whether piceatannol reduces BP and HR or whether it is associated with inhibition of RVLM and RSNA, as well as resveratrol. As obesity is closely related to insulin resistance and increased sympathetic nerve activation, piceatannol may exert beneficial effects on insulin sensitivity, BP and HR in overweight men. In addition, piceatannol has been already reported to modify cardiac protein expression such as ephrin-B1, a membrane protein that contributes to maintaining cardiomyocyte architecture, in Zucker obese rats. Therefore, a direct effect of piceatannol on cardiac muscle cannot be excluded [38]. Further studies are needed to determine the mechanism of piceatannol for glucose metabolism, BP and HR, as well as why piceatannol only showed benefits in overweight men.

In addition, polyphenol, including resveratrol and piceatannol, induces the activation of Sirt1 and AMPK [10,28,39], which leads to better metabolic health. Kawakami et al. previously reported that piceatannol and its metabolite, isorhapontigenin induce Sirt1 expression in THP-1 cells [15]. However, piceatannol supplementation did not exhibit a significant change in Sirt1 expression in PBMNCs in any group in this study.

There were several limitations to our study design. This study occurred over a short time period, and the number of participants was small. In addition, we supplemented piceatannol at a dose of 20 mg/day in humans. However, the most effective dose of piceatannol needs to be investigated in a future study.

## 5. Conclusions

Our data indicated that supplementation with piceatannol improves insulin sensitivity and might be able to reduce BP and HR in overweight men. However, piceatannol is not associated with other data, including BW, body composition, endothelial function, lipid profiles, inflammation, oxidative stress, mood status, and Sirt1 and p-AMPK expression in isolated PBMNCs. As insulin resistance is the most important factor in the pathophysiology of MS and because hypertension is also a complication of MS, supplementation with piceatannol may be useful for metabolic health, particularly for improving insulin sensitivity in obese men. Insulin resistance is closely related to aging. Therefore, piceatannol may have an anti-aging effect. However, the sample size for this study was very small (*n* = 5/group) and further studies should be conducted to determine the mechanism by which piceatannol improves insulin sensitivity, as well as reduces BP and HR, and to investigate the dose of piceatannol that is most useful to exert its benefit.

## Figures and Tables

**Table 1:** Baseline characteristics.

Characteristics	Men						Women					
	Non-Overweight			Overweight			Non-Overweight			Overweight		
	Placebo *n* = 5	Piceatannol *n* = 5	*p* value	Placebo *n* = 5	Piceatannol *n* = 5	*p* value	Placebo *n* = 5	Piceatannol *n* = 5	*p* value	Placebo *n* = 4	Piceatannol *n* = 5	*p* Value
**Age (year)**	38.8 ± 11.1	31.6 ± 4.4	0.3457	36.4 ± 5.5	32.0 ± 6.8	0.1732	38.0 ± 7.5	34.0 ± 7.6	0.2087	40.5 ± 14.5	39.8 ± 9.5	1.0000
**Body weight (kg)**	66.3 ± 3.4	64.3 ± 4.5	0.6761	79.1 ± 6.1	75.4 ± 4.3	0.2963	48.5 ± 4.9	50.4 ± 3.2	0.3457	63.6 ± 9.3	70.2 ± 7.0	0.1779
**Body mass index (kg/m^2^)**	22.2 ± 2.1	21.8 ± 1.6	1.0000	28.0 ± 0.9	26.9 ± 1.1	0.2087	20.5 ± 1.6	19.2 ± 1.5	0.1437	26.5 ± 2.7	28.0 ± 3.0	0.9009
**Systolic BP (mmHg)**	124.2 ± 11.3	119.8 ± 14.5	0.6761	120.6 ± 8.7	131.4 ± 8.9	0.1425	112.4 ± 10.7	112.4 ± 3.6	0.8237	121.0 ± 19.5	117.2 ± 22.7	0.8057
**Diastolic BP (mmHg)**	80.4 ± 12.1	68.8 ± 9.3	0.1732	77.0 ± 12.2	85.6 ± 6.5	0.2101	70.4 ± 9.8	72.8 ± 6.4	0.6723	75.0 ± 13.0	66.6 ± 9.0	0.3893
**Heart rate (/min)**	61.6 ± 12.1	62.0 ± 11.0	0.9166	58.0 ± 8.2	67.6 ± 5.3	0.6733	71.4 ± 13.4	73.6 ± 11.8	0.6004	66.5 ± 4.4	58.0 ± 10.0	0.1099
**Glucose (mg/dL)**	93.2 ± 5.6	88.4 ± 7.1	0.4005	94.2 ± 5.2	91.0 ± 14.2	0.1437	90.0 ± 6.1	81.6 ± 7.2	0.0947	96.0 ± 14.2	88.4 ± 7.7	0.3832
**Insulin (μU/mL)**	5.2 ± 3.3	3.6 ± 1.8	0.5309	6.1 ± 1.0	8.3 ± 1.9	0.0367	5.1 ± 1.7	5.5 ± 3.1	0.8345	7.5 ± 3.1	6.6 ± 2.2	0.7133
**HbA1c (%)**	5.6 ± 0.2	5.2 ± 0.2	0.0439	5.5 ± 0.1	5.4 ± 0.3	0.2017	5.4 ± 0.3	5.2 ± 0.6	1.0000	5.7 ± 0.4	5.7 ± 0.3	0.8049
**Glycated albumin (%)**	14.2 ± 0.9	13.7 ± 1.1	0.5309	12.9 ± 0.5	13.3 ± 1.9	0.9163	14.2 ± 1.2	14.4 ± 1.6	1.0000	13.5 ± 1.2	13.3 ± 0.4	1.0000
**Total cholesterol (mg/dL)**	188.8 ± 33.6	173.6 ± 15.1	0.4633	210.0 ± 43.8	243.2 ± 55.7	0.4034	181.4 ± 32.6	185.6 ± 31.0	0.6761	223.8 ± 26.5	210.8 ± 20.1	0.3893
**HDL cholesterol (mg/dL)**	59.6 ± 10.1	60.6 ± 16.3	1.0000	58.2 ± 14.4	54.4 ± 10.8	0.7526	70.6 ± 25.6	71.2 ± 8.9	0.5296	61.3 ± 12.9	54.0 ± 18.1	0.4606
**Triglyceride (mg/dL)**	85.2 ± 40.9	66.8 ± 37.2	0.4620	98.6 ± 42.9	181.0 ± 66.4	0.0367	48.8 ± 13.9	39.4 ± 10.7	0.2087	102.0 ± 29.0	92.0 ± 44.8	0.9025
**LDL cholesterol (mg/dL)**	109.8 ± 29.2	92.4 ± 9.4	0.4034	129.8 ± 28.8	151.0 ± 56.0	0.5309	93.2 ± 32.0	97.6 ± 20.8	0.8345	136.5 ± 22.8	131.2 ± 21.9	0.7133
**AST (U/L)**	22.6 ± 6.6	18.2 ± 2.6	0.2031	22.0 ± 6.7	34.4 ± 21.1	0.2477	15.4 ± 0.9	19.4 ± 6.7	0.2343	26.3 ± 10.4	17.6 ± 2.3	0.3873
**ALT (U/L)**	27.8 ± 22.2	16.4 ± 6.2	0.3443	31.8 ± 9.9	50.4 ± 31.3	0.2087	12.6 ± 2.4	18.8 ± 12.3	0.4633	34.5 ± 18.2	20.0 ± 8.2	0.1779
**γ-GTP (U/L)**	32.6 ± 14.8	29.4 ± 27.2	0.5309	74.6 ± 73.6	106.2 ± 99.6	0.2101	19.8 ± 6.8	17.0 ± 6.3	0.4578	38.8 ± 22.3	18.2 ± 6.5	0.1400
**Creatinine (mg/dL)**	0.9 ± 0.1	0.8 ± 0.1	0.3398	0.8 ± 0.0	0.8 ± 0.1	1.0000	0.6 ± 0.0	0.6 ± 0.0	1.0000	0.6 ± 0.1	0.6 ± 0.1	0.8057
**eGFR (mL/min/1.73 m^2^)**	77.2 ± 11.2	88.4 ± 11.1	0.2101	85.3 ± 6.7	89.8 ± 9.9	1.0000	85.2 ± 9.3	88.8 ± 9.7	0.5309	86.6 ± 23.9	88.0 ± 15.3	0.7133
**Uric acid (mg/dL)**	6.5 ± 1.8	6.3 ± 0.6	0.1719	6.7 ± 1.1	6.9 ± 0.6	1.0000	4.1 ± 0.8	4.3 ± 0.2	0.6761	5.1 ± 1.3	4.8 ± 0.4	0.7133

**Table 2:** Body weight, body mass index, and body composition including % fat, muscle weight and visceral fat area, blood pressure and heart rate at baseline and after 8 weeks of placebo or piceatannol supplementation (a: men, b: women).

**a**	**Men**											
	**Non-overweight**						**Overweight**					
	**Placebo (*n* = 5)**			**Piceatannol (*n* = 5)**			**Placebo (*n* = 5)**			**Piceatannol (*n* = 5)**		
	**0 Week**	**8 Weeks**	***p***	**0 Week**	**8 Weeks**	***p***	**0 week**	**8 Weeks**	***p***	**0 Week**	**8 Weeks**	***p***
**BW (kg)**	66.3 ± 3.4	65.8 ± 4.1	0.4399	64.3 ± 4.5	64.8 ± 4.8	0.1872	79.1 ± 6.1	78.6 ± 5.9	0.3153	75.4 ± 4.3	73.9 ± 6.2	0.1724
**BMI (kg/m^2^)**	22.2 ± 2.1	22.0 ± 2.3	0.3380	21.8 ± 1.6	22.0 ± 1.5	0.2262	28.0 ± 0.9	27.8 ± 0.7	0.3472	26.9 ± 1.1	26.3 ± 1.6	0.1641
**Fat (%)**	18.6 ± 4.2	18.9 ± 4.5	0.4237	17.3 ± 2.6	17.6 ± 1.8	0.4691	28.8 ± 1.5	28.5 ± 1.7	0.5231	26.7 ± 3.7	26.7 ± 3.4	0.8915
**Muscle (kg)**	30.3 ± 2.5	29.9 ± 2.6	0.1250	29.8 ± 1.5	29.9 ± 1.7	0.8750	31.8 ± 3.2	31.7 ± 2.8	0.625	31.3 ± 3.1	30.8 ± 3.5	0.1875
**VFA (cm^2^)**	48.4 ± 12.9	49.2 ± 15.7	0.8125	44.1 ± 13.7	45.8 ± 12.6	0.0625	92.6 ± 7.1	91.9 ± 9.2	0.8125	81.3 ± 15.6	78.5 ± 16.7	0.3125
**Systolic BP (mmHg)**	124.2 ± 11.3	121.2 ± 4.8	0.4705	119.8 ± 14.5	110.8 ± 9.6	0.2499	120.6 ± 8.7	119.2 ± 16.2	0.8029	131.4 ± 8.9	120.8 ± 14.4	0.0279
**Diastolic BP (mmHg)**	80.4 ± 12.1	71.4 ± 9.8	0.0432	68.8 ± 9.3	64.6 ± 12.4	0.1960	77.0 ± 12.2	71.0 ± 15.8	0.1278	85.6 ± 6.5	77.0 ± 11.9	0.0304
**Heart rate (/min)**	61.6 ± 12.1	66.0 ± 11.3	0.1302	62.0 ± 11.0	58.0 ± 11.5	0.2179	58.0 ± 8.2	55.2 ± 7.7	0.3182	67.6 ± 5.3	59.0 ± 7.9	0.0360
**b**	**Women**											
	**Non-overweight**						**Overweight**					
	**Placebo (*n* = 5)**			**Piceatannol (*n* = 5)**			**Placebo (*n* = 4)**			**Piceatannol (*n* = 5)**		
	**0 Week**	**8 Weeks**	***p***	**0 Week**	**8 Weeks**	***p***	**0 Week**	**8 Weeks**	***p***	**0 Week**	**8 Weeks**	***p***
**BW (kg)**	48.5 ± 4.9	48.7 ± 5.4	0.7267	50.4 ± 3.2	49.9 ± 3.3	0.1979	63.6 ± 9.3	63.1 ± 8.7	0.6288	70.2 ± 7.0	70.2 ± 6.4	0.8806
**BMI (kg/m^2^)**	20.5 ± 1.6	20.6 ± 1.7	0.7717	19.2 ± 1.5	18.9 ± 1.4	0.1698	26.5 ± 2.7	26.3 ± 2.5	0.6430	28.0 ± 3.0	28.0 ± 2.8	0.6974
**Fat (%)**	27.0 ± 5.7	27.0 ± 5.3	1.0000	22.7 ± 4.4	21.3 ± 3.5	0.0791	37.9 ± 2.5	37.2 ± 3.0	0.1008	41.2 ± 2.6	41.4 ± 3.2	0.7438
**Muscle (kg)**	18.9 ± 1.9	18.9 ± 1.7	0.6875	21.0 ± 1.2	21.2 ± 1.3	0.7500	21.4 ± 3.6	21.4 ± 3.4	0.7500	22.3 ± 2.4	22.2 ± 2.3	0.6875
**VFA (cm^2^)**	57.2 ± 21.5	57.0 ± 22.7	1.0000	48.3 ± 11.3	45.0 ± 10.6	0.0625	118.7 ± 22.5	114.1 ± 24.7	0.3750	149.2 ± 25.8	149.2 ± 28.7	1.0000
**Systolic BP (mmHg)**	112.4 ± 10.7	113.2 ± 9.2	0.9156	112.4 ± 3.6	113.2 ± 8.3	0.8648	121.0 ± 19.5	116.8 ± 14.8	0.4787	117.2 ± 22.7	113.0 ± 14.9	0.6587
**Diastolic BP (mmHg)**	70.4 ± 9.8	70.6 ± 4.6	0.9686	72.8 ± 6.4	69.8 ± 8.0	0.4734	75.0 ± 13.0	72.8 ± 14.6	0.3910	66.6 ± 9.0	68.0 ± 6.7	0.5940
**Heart rate (/min)**	71.4 ± 13.4	69.4 ± 7.8	0.6046	73.6 ± 11.8	63.6 ± 3.8	0.1925	66.5 ± 4.4	67.0 ± 7.0	0.8273	58.0 ± 10.0	59.8 ± 9.0	0.3456

**Table 3:** Glucose metabolism-related data, including fasting glucose, insulin, homeostasis model assessment–insulin resistance (HOMA-IR), HbA1c and glycated albumin at baseline and 8 weeks after placebo or piceatannol supplementation (a: men, b: women). c: change rate of fasting glucose, insulin, HOMA-IR, HbA1c and glycated albumin for 8 weeks of placebo or piceatannol supplementation.

**a**	**Men**											
	**Non-Overweight**						**Overweight**					
	**Placebo (*n* = 5)**			**Piceatannol (*n* = 5)**			**Placebo (*n* = 5)**			**Piceatannol (*n* = 5)**		
	**0 Week**	**8 Weeks**	***p***	**0 Week**	**8 Weeks**	***p***	**0 Week**	**8 Weeks**	***p***	**0 Week**	**8 Weeks**	***p***
**Glucose (mg/dL)**	93.2 ± 5.6	94.0 ± 8.6	0.7174	88.4 ± 7.1	87.0 ± 6.0	0.6437	94.2 ± 5.2	97.8 ± 5.0	0.1561	91.0 ± 14.2	90.8 ± 8.8	0.9515
**Insulin (μU/mL)**	5.2 ± 3.3	4.7 ± 2.1	0.5821	3.6 ± 1.8	3.9 ± 1.0	0.7931	6.1 ± 1.0	7.7 ± 2.9	0.1918	8.3 ± 1.9	6.7 ± 1.4	0.0244
**HOMA-IR**	1.2 ± 0.8	1.1 ± 0.6	0.6250	0.8 ± 0.4	0.8 ± 0.2	1.0000	1.4 ± 0.2	1.9 ± 0.8	0.1250	1.9 ± 0.8	1.5 ± 0.5	0.0625
**HbA1c (%)**	5.6 ± 0.2	5.5 ± 0.2	0.1250	5.2 ± 0.2	5.2 ± 0.2	0.7500	5.5 ± 0.1	5.4 ± 0.1	0.2500	5.4 ± 0.3	5.5 ± 0.2	0.6250
**Glycated albumin (%)**	14.2 ± 0.9	14.1 ± 0.9	0.7753	13.7 ± 1.1	13.5 ± 1.0	0.4113	12.9 ± 0.5	12.6 ± 0.7	0.1404	13.3 ± 1.9	13.1 ± 2.0	0.2560
**b**	**Women**											
	**Non-overweight**						**Overweight**					
	**Placebo (*n* = 5)**			**Piceatannol (*n* = 5)**			**Placebo (*n* = 4)**			**Piceatannol (*n* = 5)**		
	**0 Week**	**8 Weeks**	***p***	**0 Week**	**8 Weeks**	***p***	**0 Week**	**8 Weeks**	***p***	**0 Week**	**8 Weeks**	***p***
**Glucose (mg/dL)**	90.0 ± 6.1	88.6 ± 6.2	0.3111	81.6 ± 7.2	82.8 ± 4.3	0.7624	96.0 ± 14.2	95.0 ± 12.5	0.4740	88.4 ± 7.7	90.2 ± 6.2	0.4211
**Insulin (μU/mL)**	5.1 ± 1.7	5.6 ± 1.2	0.5578	5.5 ± 3.1	5.7 ± 1.9	0.9017	7.5 ± 3.1	7.2 ± 3.0	0.8859	6.6 ± 2.2	7.4 ± 1.1	0.2789
**HOMA-IR**	1.1 ± 0.3	1.2 ± 0.3	1.0000	1.1 ± 0.5	1.2 ± 0.4	0.8750	1.9 ± 1.0	1.7 ± 0.8	0.8750	1.5 ± 0.5	1.7 ± 0.2	0.2500
**HbA1c (%)**	5.4 ± 0.3	5.6 ± 0.3	0.1250	5.2 ± 0.6	5.1 ± 0.6	0.5000	5.7 ± 0.4	5.7 ± 0.3	1.0000	5.7 ± 0.3	5.7 ± 0.3	0.7500
**Glycated albumin (%)**	14.2 ± 1.2	14.2 ± 1.5	0.8083	14.4 ± 1.6	13.9 ± 1.4	0.0118	13.5 ± 1.2	13.5 ± 1.0	0.6714	13.3 ± 0.4	13.3 ± 0.4	0.9062
**c**	**Men**											
	**Non-overweight**						**Overweight**					
	**Placebo (*n* = 5)**		**Piceatannol (*n* = 5)**		***p***		**Placebo (*n* = 5)**		**Piceatannol (*n* = 5)**		***p***	
**Glucose (%)**	0.8 ± 4.8		−1.3 ± 7.2		0.6761		3.9 ± 4.9		0.5 ± 6.9		0.5309	
**Insulin (%)**	5.7 ± 43.5		33.8 ± 77.6		0.6761		23.7 ± 31.8		−18.8 ± 11.2		0.0216	
**HOMA-IR (%)**	5.4 ± 47.6		26.7 ± 67.3		0.9166		31.4 ± 39.7		−17.2 ± 11.5		0.0160	
**HbA1c (%)**	−1.8 ± 1.2		1.3 ± 4.4		0.2031		−1.1 ± 1.0		1.2 ± 3.0		0.2017	
**Glycated albumin (%)**	−0.4 ± 3.2		−1.6 ± 4.2		0.5309		−2.5 ± 3.2		−1.3 ± 2.3		0.4020	

**Table 4:** Liver function tests, including aspartate aminotransferase (AST), alanine transaminase (ALT) and γ-glutamyl transpeptidase (γ-GTP), and serum creatinine (Cr), estimated GFR (glomerular filtration rate), serum uric acid (UA), and lipid data, including total cholesterol (TC), high-density lipoprotein-cholesterol (HDL), low-density lipoprotein-cholesterol (LDL), triglycerides (TG) and free fatty acid (FFA) levels at baseline and after 8 weeks of placebo or piceatannol supplementation (a: men, b: women).

**a**	**Men**											
	**Non-Overweight**						**Overweight**					
	**Placebo (*n* = 5)**			**Piceatannol (*n* = 5)**			**Placebo (*n* = 5)**			**Piceatannol (*n* = 5)**		
	**0 Week**	**8 Weeks**	***p***	**0 Week**	**8 Weeks**	***p***	**0 Week**	**8 Weeks**	***p***	**0 Week**	**8 Weeks**	***p***
**AST (U/L)**	22.6 ± 6.6	22.2 ± 7.9	0.8125	18.2 ± 2.6	19.8 ± 2.8	0.5000	22.0 ± 6.7	19.8 ± 4.7	0.2500	34.4 ± 21.1	23.4 ± 2.2	0.3125
**ALT (U/L)**	27.8 ± 22.2	28.4 ± 25.9	0.8750	16.4 ± 6.2	16.4 ± 5.0	1.0000	31.8 ± 9.9	31.0 ± 8.6	1.0000	50.4 ± 31.3	38.6 ± 13.2	0.1875
**γ-GTP (U/L)**	32.6 ± 14.8	29.0 ± 17.3	0.8125	29.4 ± 27.2	26.6 ± 20.5	0.7500	74.6 ± 73.6	66.4 ± 54.0	0.4375	106.2 ± 99.6	89.2 ± 78.1	0.1250
**Cr (mg/dL)**	0.9 ± 0.1	0.9 ± 0.1	0.6503	0.8 ± 0.1	0.9 ± 0.1	0.5769	0.8 ± 0.0	0.8 ± 0.1	0.4543	0.8 ± 0.1	0.8 ± 0.1	0.3739
**eGFR (mL/min/1.73 m^2^)**	77.2 ± 11.2	75.5 ± 13.4	0.6176	88.4 ± 11.1	86.4 ± 8.7	0.6044	85.3 ± 6.7	87.1 ± 8.1	0.4120	89.8 ± 9.9	89.2 ± 11.5	0.5121
**UA (mg/dL)**	6.5 ± 1.8	6.6 ± 2.0	0.7349	6.3 ± 0.6	6.2 ± 0.6	0.9004	6.7 ± 1.1	6.9 ± 1.0	0.2904	6.9 ± 0.6	6.8 ± 0.6	0.4130
**TC (mg/dL)**	188.8 ± 33.6	188.6 ± 33.8	0.8750	173.6 ± 15.1	168.4 ± 20.8	0.6250	210.0 ± 43.8	210.8 ± 26.4	0.6250	243.2 ± 55.7	224.2 ± 47.9	0.0625
**HDL-C (mg/dL)**	59.6 ± 10.1	59.8 ± 9.0	0.7500	60.6 ± 16.3	58.0 ± 18.5	0.3750	58.2 ± 14.4	54.2 ± 9.4	0.3125	54.4 ± 10.8	53.4 ± 8.1	0.6875
**TG (mg/dL)**	85.2 ± 40.9	74.4 ± 31.5	0.3125	66.8 ± 37.2	61.8 ± 15.4	1.0000	98.6 ± 42.9	102.8 ± 40.9	1.0000	181.0 ± 66.4	126.4 ± 42.8	0.3125
**LDL-C (mg/dL)**	109.8 ± 29.2	109.6 ± 36.1	0.7500	92.4 ± 9.4	88.4 ± 7.3	0.6250	129.8 ± 28.8	133.6 ± 24.4	0.6250	151.0 ± 56.0	141.4 ± 39.6	0.3750
**FFA (μEq/L)**	362.4 ± 232.9	568.6 ± 104.6	0.2215	353.4 ± 185.1	436.0 ± 137.8	0.4916	474.2 ± 125.1	396.2 ± 130.4	0.1526	450.0 ± 103.6	532.2 ± 141.6	0.3593
**b**	**Women**											
	**Non-Overweight**						**Overweight**					
	**Placebo (*n* = 5)**			**Piceatannol (*n* = 5)**			**Placebo (*n* = 4)**			**Piceatannol (*n* = 5)**		
	**0 Week**	**8 Weeks**	***p***	**0 Week**	**8 Weeks**	***p***	**0 Week**	**8 Weeks**	***p***	**0 Week**	**8 Weeks**	***p***
**AST (U/L)**	15.4 ± 0.9	15.4 ± 2.4	1.0000	19.4 ± 6.7	23.6 ± 11.1	0.3750	26.3 ± 10.4	21.3 ± 6.3	0.2500	17.6 ± 2.3	19.4 ± 5.8	0.5000
**ALT (U/L)**	12.6 ± 2.4	13.4 ± 2.9	0.8125	18.8 ± 12.3	26.2 ± 24.3	0.8750	34.5 ± 18.2	25.5 ± 7.0	0.6250	20.0 ± 8.2	22.6 ± 9.7	0.2500
**γ-GTP (U/L)**	19.8 ± 6.8	25.6 ± 12.1	0.2500	17.0 ± 6.3	14.2 ± 3.6	0.6875	38.8 ± 22.3	35.8 ± 28.3	0.6250	18.2 ± 6.5	19.4 ± 5.4	0.3750
**Cr (mg/dL)**	0.6 ± 0.0	0.6 ± 0.0	0.1281	0.6 ± 0.0	0.6 ± 0.1	0.4530	0.6 ± 0.1	0.6 ± 0.1	0.4228	0.6 ± 0.1	0.6 ± 0.1	0.1668
**eGFR (mL/min/1.73 m^2^)**	85.2 ± 9.3	89.4 ± 10.4	0.1330	88.8 ± 9.7	93.0 ± 15.3	0.3403	86.6 ± 23.9	89.2 ± 28.3	0.3479	88.0 ± 15.3	83.1 ± 15.8	0.2376
**UA (mg/dL)**	4.1 ± 0.8	4.1 ± 0.5	0.9375	4.3 ± 0.2	3.8 ± 0.9	0.3665	5.1 ± 1.3	5.0 ± 1.4	0.6447	4.8 ± 0.4	4.8 ± 0.4	0.8390
**TC (mg/dL)**	181.4 ± 32.6	175.8 ± 19.1	0.6250	185.6 ± 31.0	187.0 ± 29.5	1.0000	223.8 ± 26.5	207.3 ± 17.1	0.3750	210.8 ± 20.1	213.4 ± 16.4	0.8125
**HDL-C (mg/dL)**	70.6 ± 25.6	66.0 ± 21.6	0.4375	71.2 ± 8.9	68.4 ± 8.3	0.1875	61.3 ± 12.9	54.8 ± 9.7	0.1250	54.0 ± 18.1	53.0 ± 13.5	0.7500
**TG (mg/dL)**	48.8 ± 13.9	55.6 ± 17.5	0.3125	39.4 ± 10.7	44.2 ± 9.5	0.4375	102.0 ± 29.0	102.5 ± 48.0	0.8750	92.0 ± 44.8	114.6 ± 66.3	0.1875
**LDL-C (mg/dL)**	93.2 ± 32.0	89.2 ± 30.4	0.6250	97.6 ± 20.8	102.6 ± 20.7	0.1250	136.5 ± 22.8	126.8 ± 19.2	0.2500	131.2 ± 21.9	131.0 ± 22.6	0.8125
**FFA (μEq/L)**	541.4 ± 142.4	774.6 ± 251.9	0.2217	543.2 ± 287.4	743.4 ± 158.1	0.1474	481.5 ± 110.6	588.3 ± 78.6	0.2474	485.8 ± 141.2	619.4 ± 147.3	0.2985

**Table 5:** Flow Mediated Dilation (FMD), asymmetric dimethylarginine (ADMA), high sensitive C-reactive protein (hsCRP), interleukin-6 (IL-6), diacron reactive oxygen metabolite (dROM) and biological antioxidant potential (BAP) at baseline and after 8 weeks of placebo or piceatannol supplementation (a: men, b: women).

**a**	**Men**											
	**Non-Overweight**						**Overweight**					
	**Placebo (*n* = 5)**			**Piceatannol (*n* = 5)**			**Placebo (*n* = 5)**			**Piceatannol (*n* = 5)**		
	**0 Week**	**8 Weeks**	***p***	**0 Week**	**8 Weeks**	***p***	**0 Week**	**8 Weeks**	***p***	**0 Week**	**8 Weeks**	***p***
**FMD (%)**	7.0 ± 2.3	6.6 ± 2.1	0.8501	6.6 ± 4.8	8.4 ± 2.7	0.6037	7.5 ± 4.0	7.9 ± 3.9	0.7589	5.8 ± 3.9	5.1 ± 3.0	0.5940
**ADMA (nmol/mL)**	0.39 ± 0.04	0.40 ± 0.05	0.5734	0.37 ± 0.04	0.37 ± 0.03	0.7174	0.38 ± 0.04	0.40 ± 0.05	0.2205	0.37 ± 0.03	0.37 ± 0.05	0.7449
**hsCRP (ng/mL)**	597.8 ± 630.0	216.4 ± 141.9	0.4375	215.0 ± 105.8	627.8 ± 580.4	0.0625	2334.8 ± 3704.5	843.8 ± 459.5	0.6250	1086.4 ± 751.9	2255.4 ± 3869.1	1.0000
**IL-6 (pg/mL)**	1.22 ± 1.41	1.32 ± 1.90	0.8750	0.68 ± 0.31	0.90 ± 0.37	0.1250	1.76 ± 0.72	1.18 ± 0.38	0.1875	0.92 ± 0.08	0.80 ± 0.14	0.3125
**d-ROM (U. Carr)**	298.4 ± 94.5	266.2 ± 68.8	0.1546	275.6 ± 15.1	266.8 ± 60.9	0.7700	271.8 ± 66.3	268.4 ± 66.8	0.8592	317.8 ± 43.3	317.6 ± 89.0	0.9938
**BAP (pmol/L)**	2391.2 ± 317.3	2272.8 ± 195.8	0.3642	2250.8 ± 322.5	2276.6 ± 314.2	0.8604	2376.8 ± 214.5	2356.6 ± 238.6	0.8615	2136.2 ± 218.3	2385.8 ± 419.9	0.3506
**b**	**Women**											
	**Non-Overweight**						**Overweight**					
	**Placebo (*n* = 5)**			**Piceatannol (*n* = 5)**			**Placebo (*n* = 4)**			**Piceatannol (*n* = 5)**		
	**0 Week**	**8 Weeks**	***p***	**0 Week**	**8 Weeks**	***p***	**0 Week**	**8 Weeks**	***p***	**0 Week**	**8 Week**	***p***
**FMD (%)**	7.5 ± 1.6	7.8 ± 3.3	0.8795	8.0 ± 2.9	8.3 ± 3.6	0.7764	4.8 ± 2.1	5.8 ± 2.8	0.2860	6.8 ± 3.5	9.4 ± 2.1	0.0921
**ADMA (nmol/mL)**	0.36 ± 0.04	0.35 ± 0.03	0.3739	0.36 ± 0.05	0.36 ± 0.04	1.0000	0.45 ± 0.04	0.43 ± 0.03	0.6223	0.40 ± 0.06	0.40 ± 0.07	0.9264
**hsCRP (ng/mL)**	893.0 ± 1698.3	746.8 ± 671.0	0.6250	212.6 ± 233.1	117.6 ± 56.9	0.1250	1405.0 ± 1543.2	955.8 ± 953.8	0.3750	709.2 ± 703.7	889.8 ± 604.0	0.1875
**IL-6 (pg/mL)**	0.64 ± 0.24	1.30 ± 1.21	0.2500	0.92 ± 0.28	0.94 ± 0.21	1.0000	1.68 ± 0.59	1.37 ± 0.69	0.3750	1.50 ± 0.41	1.32 ± 0.38	0.6250
**d-ROM (U. Carr)**	349.8 ± 54.7	338.6 ± 20.7	0.6233	354.2 ± 37.8	308.6 ± 30.7	0.1607	349.3 ± 51.8	343.5 ± 10.8	0.8523	332.4 ± 31.8	362.4 ± 20.1	0.1058
**BAP (pmol/L)**	2359.2 ± 287.9	2407.6 ± 246.9	0.7853	2486.8 ± 182.9	2120.0 ± 207.7	0.0413	2151.0 ± 62.8	2135.8 ± 293.2	0.9202	2299.2 ± 178.5	2378.6 ± 430.6	0.6406

## Supplementary Materials

The following are available online at www.mdpi.com/2072-6643/9/10/1142/s1, Figure S1. The graphs show the change rate of Sirt1/β-actin and phospho(p)-AMPK/AMPK in isolated peripheral mononuclear cells for 8 weeks of intervention, which were evaluated by western blotting, Table S1. Data of the visual analogue scale (VAS) and the Profile of Mood States (POMS), including scores of “tension”, “depression”, “anger”, “fatigue”, “confusion” and “vigor” and total mood disturbance score at baseline and after 8 weeks of placebo or piceatannol supplementation (a: men, b: women), Table S2. Adverse events during the intervention.
