# Supplementary material for: The Effect of Piceatannol from Passion Fruit (Passiflora edulis) Seeds on Metabolic Health in Humans
Source: Nutrients. 2017 Oct 18;9(10):1142. doi: 10.3390/nu9101142 (PMC5691758; doi:10.3390/nu9101142)
Supplement: Supplementary file 1 [file nutrients-09-01142-s001.zip › nutrients-225260-supplementary.pdf]

nutrients

MDPI

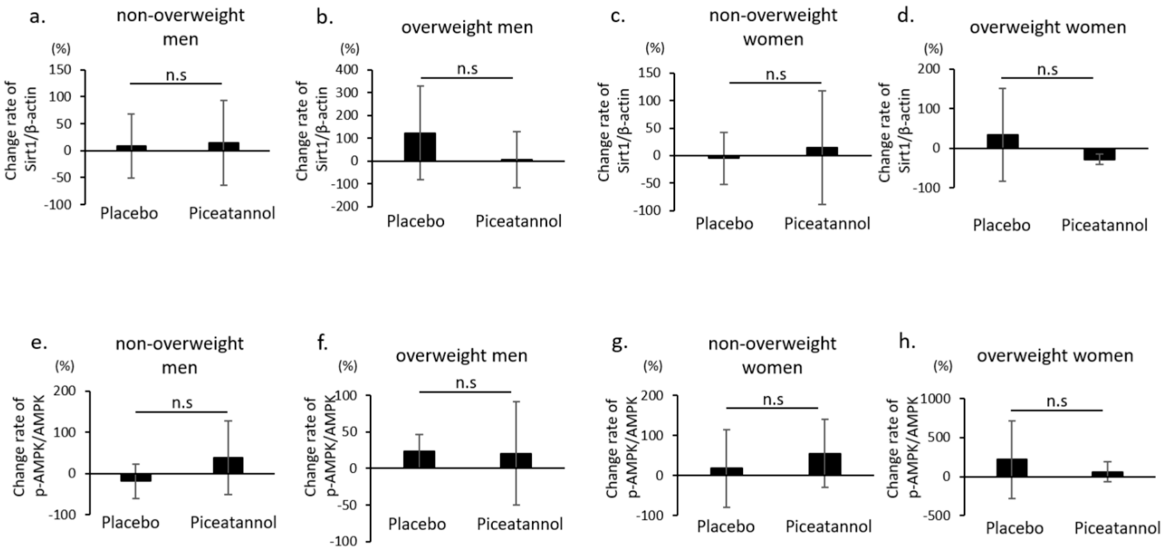

Figure S1. The graphs show the change rate of Sirt1/β-actin and phospho(p)-AMPK/AMPK in isolated peripheral mononuclear cells for 8 weeks of intervention, which were evaluated by western blotting (a and b: Sirt1/β-actin, e and f: p-AMPK/AMPK in non-overweight or overweight men. c and d: Sirt1/β-actin, g and h: p-AMPK/AMPK in non-overweight or overweight women). The data are presented as the mean ± S.D. n.s. denotes no significance.

Table S1. Data of the visual analogue scale (VAS) and the Profile of Mood States (POMS), including scores of 'tension', 'depression', 'anger', 'fatigue', 'confusion' and 'vigor' and total mood disturbance score at baseline and after 8 weeks of placebo or piceatannol supplementation (a: men, b: women).

a.	Men											
	Non-overweight						Overweight					
	Placebo (n=5)			Piceatannol (n=5)			Placebo (n=5)			Piceatannol (n=5)		
	0w	8w	p	0w	8w	p	0w	8w	p	0w	8w	p
VAS (cm)	3.0±1.9	4.9±2.2	0.6250	3.1±1.4	4.1±1.7	0.8750	4.5±2.2	4.5±2.4	1.0000	2.9±0.9	3.2±1.7	0.4375
POMS- Tension	43.0±9.1	46.0±9.8	0.8125	39.4±10.5	39.3±5.5	1.0000	44.3±12.4	41.1±9.6	0.2500	41.6±4.2	42.6±3.8	0.8750
-Depression	47.6±7.7	47.5±7.2	0.8750	40.7±1.6	42.4±4.0	0.5000	47.1±9.2	48.1±11.1	0.7500	43.9±2.1	45.8±5.3	0.3750
-Anger	44.5±7.9	43.9±6.7	1.0000	40.3±4.7	41.9±3.0	0.3750	46.2±4.5	45.7±7.8	0.7500	42.1±5.3	44.2±4.9	0.2500
-Vigour	43.6±10.6	47.9±11.4	0.5000	49.6±6.1	51.1±4.0	0.5000	54.9±9.5	46.6±9.9	0.1875	49.8±9.5	46.5±8.5	0.4375
-Fatigue	47.3±8.9	46.5±6.9	0.8750	39.9±7.4	42.1±5.7	0.8750	50.9±13.1	49.5±11.8	0.6875	46.1±7.4	48.6±7.9	0.6250
-Confusion	49.1±6.3	46.4±7.2	0.5000	41.8±4.6	41.3±4.4	1.0000	42.2±9.5	46.2±6.5	0.3750	44.7±4.0	45.9±6.4	0.5000
Total	187.9±28.2	182.4±40.5	1.0000	152.6±28.6	155.9±20.5	0.8125	175.8±48.2	184.1±46.8	0.6250	168.6±23.1	180.6±28.0	0.1875

b.	Women											
	Non-overweight						Overweight					
	Placebo (n=5)			Piceatannol (n=5)			Placebo (n=4)			Piceatannol (n=5)		
	0w	8w	p	0w	8w	p	0w	8w	p	0w	8w	p
VAS (cm)	3.2±1.5	4.4±2.3	0.3125	2.6±0.7	2.6±1.1	1.0000	3.1±2.0	2.0±1.5	0.2500	4.3±2.0	4.1±2.1	1.0000
POMS- Tension	41.1±6.2	39.5±4.9	0.5000	42.6±7.9	41.4±7.2	0.8125	44.0±7.0	46.3±4.6	0.5000	51.2±7.2	52.4±12.0	1.0000
-Depression	43.1±4.1	41.9±4.3	0.7500	48.9±8.1	47.3±11.0	0.6250	48.3±9.3	48.3±5.9	1.0000	48.0±8.6	48.4±7.9	1.0000
-Anger	45.6±7.5	46.8±7.7	0.7500	47.4±8.5	49.5±5.7	0.6250	44.3±8.3	44.3±6.4	1.0000	50.4±13.6	47.3±10.5	0.2500
-Vigour	44.0±9.9	43.0±10.1	1.0000	40.4±9.9	40.9±7.9	0.7500	41.6±4.2	45.2±8.6	0.5000	43.4±3.6	42.4±4.2	0.5000
-Fatigue	45.8±1.7	46.5±4.2	0.6875	46.1±10.3	44.6±4.8	1.0000	45.1±8.8	45.7±7.2	1.0000	51.6±10.6	54.9±14.3	0.6250
-Confusion	53.0±2.5	51.7±4.2	1.0000	51.8±5.6	50.9±7.5	0.9375	52.0±4.5	56.1±5.3	0.5000	62.7±10.7	62.9±15.1	1.0000
Total	3.2±1.5	4.4±2.3	0.3125	2.6±0.7	2.6±1.1	1.0000	3.1±2.0	2.0±1.5	0.2500	4.3±2.0	4.1±2.1	1.0000

Table S2. Adverse events during the intervention.

Adverse events	Placebo (n=19)		Piceatannnol (n=20)		χ^2 (p value)
	n	n (%)	n	n (%)	
Total	9	7(36.8%)	11	7(35.0%)	1.0000
Constipation	1	1(5.3%)	1	1(5.0%)	1.0000
Peripheral edema	0	0(0.0%)	1	1(5.0%)	1.0000
Liver dysfunction	0	0(0.0%)	1	1(5.0%)	1.0000
Seasonal allergy	1	1(5.3%)	0	0(0.0%)	0.9793
Gastroenteritis	2	2(10.5%)	1	1(5.0%)	0.9631
Viral upper respiratory tract infection	4	4(21.1%)	1	1(5.0%)	0.3079
influenza	0	0(0.0%)	1	1(5.0%)	1.0000
Contusion	0	0(0.0%)	1	1(5.0%)	1.0000
Dyslipidemia	0	0(0.0%)	1	1(5.0%)	1.0000
Hyperuricemia	1	1(5.3%)	1	1(5.0%)	1.0000
Numbness	0	0(0.0%)	2	1(5.0%)	1.0000
